# Effect of Porcine Placenta Extract Supplement on Skin Condition in Healthy Adult Women: A Randomized, Double-Blind Placebo-Controlled Study

**DOI:** 10.3390/nu12061671

**Published:** 2020-06-04

**Authors:** Masumi Nagae, Maki Nagata, Mitsuhiro Teramoto, Masayuki Yamakawa, Takahiro Matsuki, Koichiro Ohnuki, Kuniyoshi Shimizu

**Affiliations:** 1 Faculty of Agriculture, Kyushu University, 744 Motooka, Nishi-ku, Fukuoka 819-0395, Japan; makinagata0817@gmail.com (M.N.); shimizu@agr.kyushu-u.ac.jp (K.S.); 2 Healthcare Business Unit, Kasyu Industries Co., Ltd., 2-5-3 Minami-Futajima, Wakamatsu-ku, Kitakyusyu, Fukuoka 808-0109, Japan; teramoto_mitsuhiro@kasyu.co.jp (M.T.); yamakawa_masayuki@kasyu.co.jp (M.Y.); matsuki_takahiro@kasyu.co.jp (T.M.); 3 Department of Biological and Environmental Chemistry, Kinki University Kyushu, Kayanomori, Izuka, Fukuoka 820-8555, Japan; ohnuki@fuk.kindai.ac.jp

**Keywords:** porcine placenta extract, skin hydration, skin barrier function, skin elasticity, menopausal symptoms

## Abstract

Placenta extract has been used as a component of ointments for skin dryness and beautification. However, little is known about the effect of oral intake of placenta extract on skin condition. The current study aimed to clinically explore the effect of oral intake of porcine placenta extract on human skin quality. A randomized controlled double-blind trial was performed on healthy women aged 40–59 years (n = 20), who were randomly assigned to receive either placebo or 200 mg of porcine placenta extract once daily for 4 weeks from 28 January 2019 to 25 February 2019. Skin quality parameters and the Simplified Menopausal Index (SMI) were assessed at baseline and after 4 weeks. After 4 weeks, three parameters of skin quality were significantly improved in the porcine placenta group compared with the placebo group. These results suggest that porcine placenta extract can be used as a health food ingredient to maintain humans’ skin condition in the dry winter season.

## 1. Introduction

The placenta is a temporary organ in the females of mammals during gestation that connects the developing fetus via the umbilical cord to the uterine wall. It is considered a reservoir of cytokines, hormones, bioactive peptides, enzymes, growth factors, vitamins, and minerals [1]. Mammalian placenta extract has been used in Chinese and Korean traditional medicines for wound healing [2,3]. Recently, benefits of the topical use of porcine placenta extract on chronic and non-healing wounds have been reported [4,5]. It is known that fibroblast growth factors (FGFs) and transforming growth factors (TGFs) are two key factor families involved in wound healing. Porcine placenta extract has been shown to reduce the wound healing time and increase FGF and TGF levels in rat skin [6]. The potential role of porcine placenta extract in skin healing and care has been shown by observations ex vivo at the cellular level or in animal models. Equally, human placenta extract has been documented to have a growth-promoting activity on skin cells [7,8]. Dermal fibroblasts enable the synthesis of collagen, elastic fibers, and dermal hyaluronic acid [9,10]. Further, FGF, keratinocyte growth factor (KGF), and stimulators of endothelial cell growth are all present in human placenta extract [11,12]. These results suggest that oral intake of placenta extract, which includes bioactive peptides, may improve human skin hydration and elasticity and reduce wrinkling. 

On the other hand, human placenta has been used medically for symptomatic treatment and has traditionally been utilized to improve menopausal symptoms in Korea [13]. Further, the immunomodulatory and antioxidant effects of placenta extract have been demonstrated in several studies [14,15]. Porcine placenta extract reportedly has antioxidant [16] and immune activity-enhancing effects [14]. Additionally, its neuroprotective and cognition-enhancing functions in a murine menopause model have been shown [16].

Because of the natural process of skin aging, the skin of women is significantly changed through menopause [17,18,19]. In post-menopausal women, estrogen deprivation often decreases collagen synthesis and skin atrophy [20,21,22], reduces skin elasticity [20,23], and increases skin dryness [24].

Since the immune effect of porcine placenta is similar to that of human placenta, the clinical and practical use of porcine placenta extract is assumed to be safe. However, there have been few reports on the clinical effects of porcine placenta. The purpose of this study was to clinically explore the effect of oral intake of porcine placenta extract on middle-aged (40–59 years) women’s skin condition, skin barrier function (transepidermal water loss: TEWL), and skin elasticity, as well as on menopausal symptoms.

## 2. Materials and Methods

### 2.1. Supplement Preparation

Fresh porcine placenta extract powder was produced by Kashu Industries Co., Ltd. (Fukuoka, Japan). In collaboration with the Institute for Virus Research of Kyoto University, a severe technique to inactivate the virus of the porcine placenta extract has been established as a part of the manufacturing process. Moreover, the pharmaceutical-grade sanitization filter was used. The safety of the placenta extract has been certified by the Japanese Institute for Health Food Standards (JIHFS). Placenta was obtained from healthy pigs, solid (placenta tissue) and liquid (cell fluid) phases were separated, and 100% extract was collected (Product code: KHP001). Based on the pre-sampling, the best physical wellbeing dose was a 200-mg intake. The placenta extract supplement was designed to be taken in two 200 mg tablets/day; the placenta extract supplement product contained a combination of 111 mg of dextrin and 100 mg of placenta extract powder (KHP001) in one tablet. The placebo product contained only 211 mg of dextrin in one tablet.

### 2.2. Clinical Study and Ethics

The study was conducted from 28 January 2019 to 25 February 2019 as a randomized double-blind placebo-controlled clinical trial in the Laboratory of Systematic Forest and Forest Products, Science Faculty of Agriculture, Kyushu University. The clinical study included two groups with a 1:1 allocation ratio of receiving placebo tablets or placenta tablets. Stratified randomization was used in the clinical trial to reduce bias. 

The study was approved by the Kindai University Faculty of Humanity-Oriented Science and Engineering Ethics Committee (4 March 2017) and was registered in the University Hospital Medical Information Network Clinical Trials registry (UMIN-CTR) with ID: 000036955.

### 2.3. Participants and Setting

Since we conducted exploratory research and there were no previous studies on the oral intake of placenta that had reached statistical significance, we referred to a previous investigation that had detected significant differences after the oral intake of another supplement [25]. A priori analysis showed that to detect an 80% difference in TEWL with alpha of 0.05, after 4 weeks of supplementation, we needed at least 8 to 11 subjects in each group. Healthy women volunteers aged 40–59 years invited via the network of the laboratory (n = 20) and were checked for eligibility with inclusion and exclusion criteria (Table 1) by a staff member (not the investigator). The women volunteers signed an informed consent form stating the purpose, method, compensation, confidentiality, and right of withdrawal from this study. In collaboration with two clinics, we could consult medical doctors in the case of an adverse event.

### 2.4. Randomization

The randomization was centralized and performed based on a computer-generated list of random numbers by a staff member independent of the investigators. The randomization was conducted based on stratified random sampling with the mean age and body mass index (under 45 years and BMI under 22; under 45 years and BMI 22 or more; 45 years or more and BMI under 22; 45 years or more and BMI 22 or more). None of the investigators were aware of group assignments or were involved in the allocation.

### 2.5. Study Schedule

All participants took two tablets (orally) of their assigned study formulation once daily. To minimize any weakness of the study, all participants were required to refrain from the intake of any similar dietary supplements, quasi drugs, or medicines. They were also prohibited from using any skincare treatments, such as face masks or packs and massages, or from changing their daily skincare cosmetics from start to finish of the study. Each participant visited the research laboratory for assessment two times: Prior to the intake of study formulation at baseline (0 W) and after 4 weeks (4 W) of study formulation intake for efficacy measurements. The participants were requested to apply daily skin care products in the mornings of the visit days and removed the products at each visit. Measurements were taken on the left cheek (an inner position 5 cm from the lower end of the right earlobe) and left upper arm (inner side, 3 cm above the elbow). The skin region of interest was cleansed using a cleansing sheet (Bifesta Cleansing Sheet, Mandom Corporation, Osaka, Japan), wiped with cotton containing a cleansing liquid (Bifesta Face Wash, Mandom Corporation), rinsed with warm water, wiped, and dried for 20 min at stable temperature (23 ± 5 °C) and humidity conditions (50% ± 15%). 

### 2.6. Measurement of Skin Areas and Menopausal Symptoms

Measurement of hydration (arbitrary unit; a.u.) and TEWL (g/h/m^2^) were taken with a Corneometer® CM 825 and TEWAMETER® TM 300, respectively (both instruments from Courage and Khazaka, Cologne, Germany). Skin elasticity (%) was measured with a Cutometer® MPA 580 (Courage and Khazaka) based on skin suction using a probe with a negative pressure of 450 mbar, which draws the test area into the aperture of the probe. Measurements were repeated three times with 2 s of suction time followed by 2 s of relaxation time for each measurement. The obtained curves of skin deformation were analyzed with Win Cutometer® MPA software to determine the values of skin-elasticity parameters: R2 (overall elasticity of the skin), R5 (net elasticity), and R7 (the ratio of elastic recovery to total deformation). Each measurement took 1–3 min to complete, and a series of five values were obtained. The three middle values were utilized to calculate the median value. The menopausal symptoms were assessed with the Simplified Menopausal Index (SMI) [26], which is commonly used by gynecologists in Japan. In this index, a higher score means more severe symptoms.

### 2.7. Outcome

The primary outcome was a response to the oral administration of supplement with respect to skin hydration and TEWL based on the skin measurement device. The secondary outcomes were the skin elasticity change according to the skin measurement devices and menopausal symptoms assessed with SMI.

### 2.8. Statistical Analysis

SPSS (version 25.0, Chicago, IL, USA) was used to analyze the data. To compare the quantitative demographic variables of age and weight between the two groups, the independent samples *t*-test, Mann–Whitney U test and Fisher’s exact test were used. Changes in variables at the end of the study compared to those at the beginning of the study were measured with the Wilcoxon signed rank test. To compare changes in parameters between the two groups, the Mann–Whitney U test was used. Statistical significance was considered at *p* < 0.05. In light of the assumed limitations for *p* values, the effect size r was also presented in the result tables as a representative of the effect size.

## 3. Results

### 3.1. Demographic Information at Baseline

**Figure 1:**
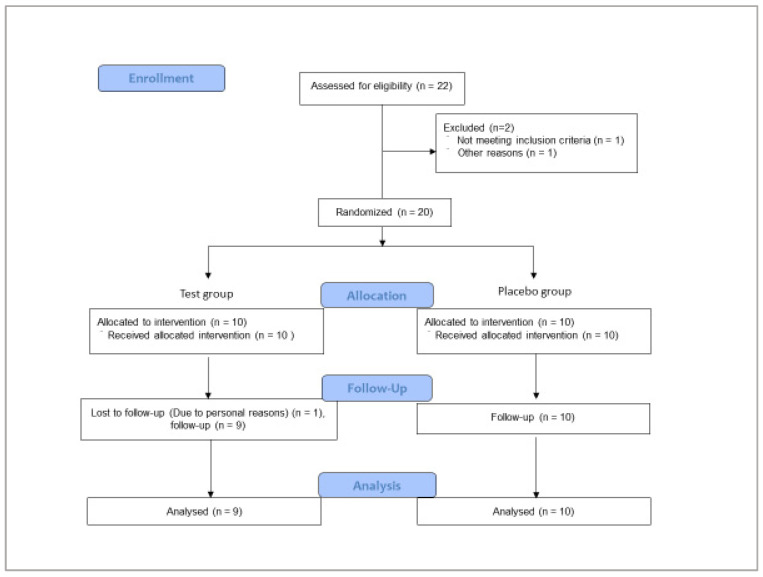
The study flow diagram.

From January 2019 to February 2019, excluding one participant who dropped out of the study for personal reasons, 19 healthy adult females completed the study (Figure 1). The background characteristics for each group are shown in Table 2. There were no significant differences between the two groups in age and body mass index. 

### 3.2. Effects of Porcine Placenta Extract on Skin Hydration and TEWL

We measured two skin sections, left cheek skin and left upper arm skin. Firstly, cheek skin hydration and TEWL were observed, with no significant differences during 4 weeks in either group. On the other hand, both arm skin hydration and TEWL were observed, with a significant difference for 4 weeks (Table 3). Secondly, we analyzed both parts’ skin data to compare the changes in parameters between the two groups (Table 4a,b). There were no different changes in skin hydration and TEWL values on the cheek between the placebo group and test group. Similarly, there were no different changes in skin hydration values on the arm between the placebo group and test group (*p* = 0.497, effect size r = −0.169). On the other hand, there was a greater difference of changes in the skin TEWL value on the arm in the placebo group than in the test group (*p* = 0.006).The effect for arm skin TEWL might be clinically significant considering the large effect size (effect size r = −0.618). 

### 3.3. Effect of Porcine Placenta Extract on Skin Elasticity

In the evaluation of skin elasticity, cheek skin R5 was observed, with a significant difference during 4 weeks in the placebo group (*p* = 0.012). On the other hand, cheek skin R2 and R5 were observed, with no significant difference during 4 weeks in either group. Arm skin R5 and R7 were observed to be significant during 4 weeks in the test group (R5; *p* = 0.028, R7; *p* < 0.001). On the other hand, arm skin R2 was observed, with no significant difference during 4 weeks in either group. Changes in arm skin elasticity R2 were significantly greater in the test group than in the placebo group (*p* = 0.017). Further, changes in arm skin elasticity R7 were significantly greater in the test group than in the placebo group (*p* = 0.003). 

### 3.4. Effect of Porcine Placenta Extract on Menopausal Symptoms

**Figure 2:**
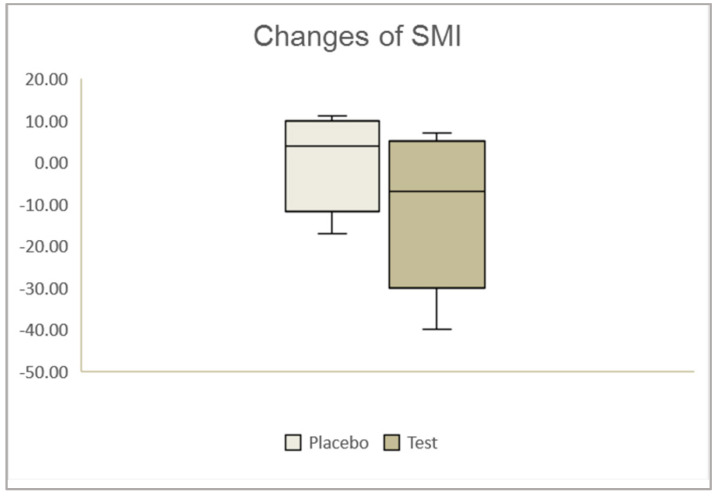
Effect of porcine placenta extract on menopausal symptoms. Comparison of changes of Simplified Menopausal Index (SMI) over 4 weeks between the placebo group and test group.

In the evaluation of menopausal symptoms, changes in SMI over 4 weeks showed a trend toward higher values in the test group than in the placebo group, although they did not reach significance (*p* = 0.079) (Figure 2).

### 3.5. Analysis of Laboratory Parameters and Adverse Reactions

Statistical analysis of the evaluated laboratory parameters showed no significant differences from their normal ranges. No adverse reactions were reported by any participant or observed by the investigator during the study.

## 4. Discussion

In the present study, we explored the effect of oral intake of porcine placenta extract on human skin condition: Skin hydration, skin barrier function (TEWL), and skin elasticity as well as on menopausal symptoms. There were more significant changes in arm skin items, such as TEWL, elasticity R2, and elasticity R7, in the test group than in the placebo group. 

The test group’s arm skin hydration increased but not clinically. However, the test group’s arm skin barrier function was maintained clinically. These observations may be a reflection of the seasonal environment and depended on whether part of skin was treated or not treated with cosmetics. The barrier function of the stratum corneum is lower in winter than in summer as the corneocytes that comprise it are smaller during the winter season than those found during the summer season [27]. This study was conducted in the dry winter season, and participants were instructed not to change cosmetics use within the 4 weeks preceding the study. A previous study reported that an increase of the hydration level and decrease of transepidermal water loss were observed after using the cosmetic for 4 weeks in both the high-hydration and low-hydration groups [28]. The effect of the placenta extract intake was apparent on part of the arm skin, which was protected by clothes but not treated with cosmetics. Moreover, it was reported that the skin surface water content of older people showed lower values than those of young people, although the TEWL values of them are similar to young people [29]. Since the sample of this study were middle-aged (40–59 years) women, the skin hydration of them might have been hard to increase than young people, but the TEWL of them might have been easy to maintain.

Our team also investigated in a cellular experiment (Nagata et al., unpublished data, see Appendix A) whether porcine placenta extract promotes the expression of ceramide synthase, which is important for maintaining the skin barrier function [30]. In human keratinocyte cells, the levels of two kinds of ceramide synthase (CERS) were higher in placenta extract-treated cells than in control cells. The expression was similar among cells treated with different concentrations of porcine placenta extract. Our results suggest that porcine placenta extract has the potential to enhance the expression of ceramide synthase genes and exhibits no cytotoxicity toward human keratinocyte cells. The barrier function of the stratum corneum is generally determined by three factors: The size of corneocytes and the number of layers of the cells, the intercellular substances that constitute the lamella structure of the stratum corneum, and parameters related to the lipid film on the skin surface [27]. These factors vary from season to season [31,32,33,34,35,36,37,38]. Since intercellular lipid lamellar structures have a water-holding capability and barrier function, promotion of the expression of ceramide synthase might maintain the skin barrier function. The intake of porcine placenta extract might have promoted the expression of ceramide synthase and maintained the skin barrier function for 4 weeks. Skin elasticity, resilience, and toughness are mainly affected by collagen and elastic-fiber networks in the extracellular matrix (ECM) of the dermis. Skin hydration also strongly affects skin elasticity, because conformational changes in elastin occur in hydrated protein [39]. The skin hydration changes may have contributed to the increase in skin elasticity. The intake of porcine placenta extract affected arm skin hydration, and subsequently, the elasticity R2 and elasticity R7 in the test group changed significantly more at 4 weeks than in the placebo group. Porcine placenta extract has potential as an anti-wrinkling agent due to its reported anti-wrinkle [40] and elastase inhibitor functions [41]. In the present study, the existence of an elastase inhibitor in porcine placenta was reported as another potential mechanism behind the increased skin elasticity. Several previous studies reported that dermal fibroblasts exist in placenta extract [11,12], whereas other investigations demonstrated that dermal fibroblasts enable the synthesis of collagen, elastic fibers, and dermal hyaluronic acid [9,10]. Although the internal mechanism of oral supplementation with porcine placenta has been unclear, a previous study reported that major porcine placenta peptides in a living organism: Glycyl-L-Leucine (Gly-Leu), L-Leucyl-Glycine (Leu-Gly), and L-Leucyl-L-Leucine (Leu-Leu), affect skin hydration and elasticity in in vitro and in vivo studies [42]. Our findings are consistent with those of previous investigations that explain the placenta extract effects with several potential mechanisms. Regarding the effect of porcine placenta extract on menopausal symptoms, although the total SMI index did not show significant changes in the placebo group, a trend toward a decrease in those parameters was shown in the test group after 4 weeks of placenta extract intake. The mean of the total SMI before supplement intake was moderate in the test group, whereas after treatment, it improved to mild. The previous study conducted with 50 climacteric Japanese women for 12 weeks reported that porcine placenta extract did not ameliorate the hormonal balance itself but improved the subjective wellbeing of climacteric women [43]. As in this study, the sample number was low, and the sample group was not explicitly diagnosed with menopause, thus it was not possible to determine whether the improvement was really reflected a change of the menopausal symptoms or the low number of samples. However, the trend of the data of improved SMI for 4 weeks apparently showed that a substantial number of participants in the test group improved their physical and mental symptoms by supplement intake.

This study demonstrates that porcine placenta supplement is useful for maintaining skin condition in the dry winter season. Porcine placenta is relatively safe, and its immune effect is similar to that of human origin, which makes porcine placenta widely usable as a cosmetic agent. To the best of our knowledge, this is the first study to clinically evaluate the effect of oral intake of porcine placenta extract on skin condition. This study has a few limitations. Since this study investigated a limited age group of 40–59 years, and the sample number was low, these results should be cautiously applied in clinical practice. Moreover, as staff without a medical license assessed the eligibility and adverse events, we could not completely exclude the underestimation or misclassification of adverse effects. Further studies including both male and female participants of different ages, whose eligibility and adverse events are assessed by medical staff, with different doses and intake periods are needed. Additionally, we will conduct further research to investigate the expression of major skin-related genes, Filaggrin, Profilaggrin, and serine palmitoyltransferase, or to evaluate the biological markers related to the well-being of the skin, such as inflammatory cytokines. Moreover, we should isolate growth factor-stimulating compounds from porcine placenta and clarify the mechanisms of action.

## Figures and Tables

**Table 1:** Inclusion and exclusion criteria.

**Inclusion criteria**
•Persons who are generally judged as healthy
•Persons who give voluntary written consent to participate in the present trial
**Exclusion criteria**
•Persons who take any dietary supplements, quasi drugs, or medicines, which cause the same or similar effects as the supplements evaluated in this study
•Persons who have changed their habits in respect to supplements or cosmetics use within the past 4 weeks
•Persons who work in night shift or in day and night shift
•Persons who have been treated for their condition or prevention in a clinic with their informed consent
•Persons with the following medical histories: skin disease or atopic dermatitis, serious diseases of sugar metabolism, lipid metabolism, hepatic function, renal function, heart, circulatory, respiratory, endocrine, or immune system, or mental illness of the nervous system
•Persons with a medical history of alcoholism or drug addiction
•Persons who may develop an allergic reaction to food
•Persons who are pregnant, breast-feeding, or hope to be pregnant during the study period
•Persons who are participating in or will participate in any other clinical trial (on the use of foods/medicines/quasi medicines/medical devices)
•Persons who are not judged suitable for participation by the investigator

**Table 2:** Background characteristics of the participants.

Item	Group	Observed Value		*p* Value
Number	Placebo	10		Not applicable
	Test	9		
Age (years)	Placebo	44.7 ± 3.4		0.851 ^a^
	Test	45.0 ± 3.4		
BMI (kg/m^2^)	Placebo	22.5 ± 3.3		0.992 ^a^
	Test	22.4 ± 2.8		
Hydration at the cheek (a.u.)	Placebo	47.8	(39.4–52.8)	0.028 ^b^
	Test	35.5	(33.4–44.4)	
Hydration at the arm (a.u.)	Placebo	20.5	(16.7–25.8)	0.905 ^b^
	Test	23.6	(16.1–25.2)	
TEWL at the cheek (g/h/m^2^)	Placebo	17.9	(15.5–26.2)	0.549 ^b^
	Test	18.3	(12.0–20.5)	
TEWL at the arm (g/h/m^2^)	Placebo	9.9	(6.2–11.6)	0.356 ^b^
	Test	10.9	(8.9–13.2)	
Menopause	Placebo	0 (0.0)		0.474 ^c^
	Test	1 (11.1)		
Irregular menstruation	Placebo	2 (20.0)		1.000 ^c^
	Test	1 (12.5)		
SMI	Placebo	31.5	(18.0–44.0)	0.065 ^b^
	Test	44.0	(31.5–68.0)	

Each value is expressed as mean ± SD or median (0.25–0.75) or n (%). *p* values were determined by ^a^ independent *t*-test or ^b^ Mann–Whitney U test or ^c^ Fisher’s exact test. BMI: Body mass index; TEWL: Transepidermal water loss; a.u.: arbitrary unit; SMI: Simplified Menopausal Index.

**Table 3:** Comparison of skin item values pre- and post- intervention.

Item (unit)	Body Part	Group	n	Pre-Intervention		Post-Intervention		*p* Value
Hydration (a.u.)	cheek	Placebo	10	47.83	(39.40–52.80)	44.95	(37.17–48.73)	0.386
		Test	9	35.47	(33.35–44.40)	36.57	(34.00–43.23)	0.515
	arm	Placebo	10	20.48	(16.74–25.82)	22.03	(19.19–26.43)	0.575
		Test	9	23.60	(16.05–25.17)	22.80	(20.08–27.10)	0.021
TEWL (g/h/m^2^)	cheek	Placebo	10	17.90	(15.49–26.16)	18.37	(16.76–21.98)	0.799
		Test	9	18.31	(12.00–20.52)	18.23	(12.80–19.14)	0.953
	arm	Placebo	10	9.90	(6.20–11.57)	12.84	(11.32–13.85)	0.005
		Test	9	10.87	(8.94–13.15)	11.14	(8.76–12.56)	0.515
Elasticity R2 (%)	cheek	Placebo	10	0.79	(0.77–0.89)	0.85	(0.79–0.91)	0.092
		Test	9	0.82	(0.81–0.84)	0.85	(0.82–0.88)	0.085
	arm	Placebo	10	0.91	(0.90–0.93)	0.91	(0.88–0.92)	0.068
		Test	9	0.90	(0.90–0.91)	0.92	(0.90–0.93)	0.154
Elasticity R5 (%)	cheek	Placebo	10	0.65	(0.53–0.72)	0.75	(0.61–0.80)	0.012
		Test	9	0.61	(0.58–0.69)	0.64	(0.58–0.74)	0.314
	arm	Placebo	10	1.07	(1.03–1.11)	1.05	(1.04–1.11)	0.919
		Test	9	1.03	(0.97–1.13)	1.11	(1.06–1.15)	0.028
Elasticity R7 (%)	cheek	Placebo	10	0.39	(0.34–0.51)	0.43	(0.35–0.54)	0.241
		Test	9	0.42	(0.37–0.43)	0.43	(0.38–0.48)	0.093
	arm	Placebo	10	0.69	(0.67–0.71)	0.71	(0.66–0.72)	0.593
		Test	9	0.69	(0.66–0.70)	0.72	(0.69–0.73)	0.000

The data are presented as the median (0.25–0.75). *p* value, Wilcoxon signed rank test (pre- vs. post-). TEWL: Transepidermal water loss; a.u.: arbitrary unit.

**Table 4:** (**a**) Comparison of changes of cheek skin values between the placebo group and test group. (**b**) Comparison of changes of arm skin values between the placebo group and test group.

(a)						
Item	Placebo (n = 10)		Test (n = 9)		*p* Value	Effects Size r
Hydration(a.u.)	−2.18	(−18.83–3.58)	2.67	(−7.08–6.62)	0.243	−0.281
TEWL (g/h/m^2^)	0.96	(−3.06–1.96)	0.45	(−3.07–2.05)	0.780	−0.075
Elasticity R2 (%)	0.02	(0.00–0.06)	0.02	(0.00–0.06)	0.905	−0.028
Elasticity R5 (%)	0.07	(0.02–0.12)	0.04	(−0.03–0.11)	0.315	−0.235
Elasticity R7 (%)	0.03	(−0.02–0.10)	0.02	(0.00–0.07)	0.905	−0.037
(**b**)						
**Item**	**Placebo (n = 10)**		**Test (n = 9)**		***p* Value**	**Effects Size r**
Hydration(a.u.)	1.85	(−4.23–4.45)	2.73	(0.43–4.98)	0.497	−0.169
TEWL (g/h/m^2^)	2.78	(1.80–5.24)	0.29	(−0.69–1.74)	0.006	−0.618
Elasticity R2 (%)	−0.02	(−0.03–0.00)	0.01	(−0.01–0.03)	0.017	−0.535
Elasticity R5 (%)	0.00	(−0.06–0.07)	0.07	(0.04–0.12)	0.065	−0.422
Elasticity R7 (%)	0.01	(−0.02–0.02)	0.03	(0.02–0.04)	0.003	−0.648

Each value is expressed as the median (0.25–0.75). *p* values were determined by Mann–Whitney U test. TEWL: transepidermal water loss; a.u.: arbitrary unit.

**Figure A1:**
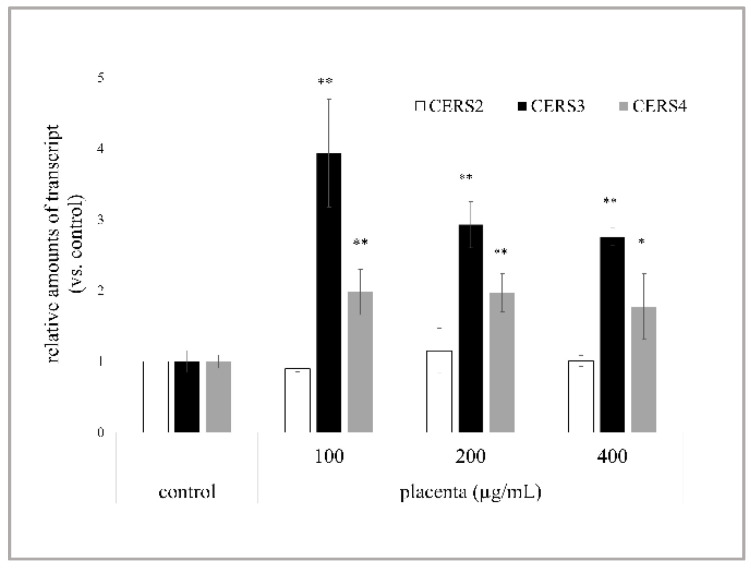
Expression of ceramide synthase in human keratinocyte cells (HaCaT) cells after porcine placenta extract application.

**Figure A2:**
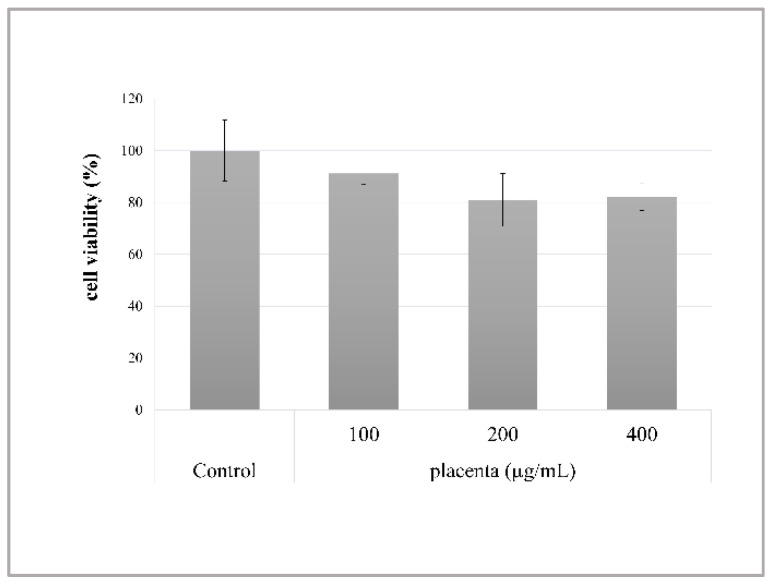
Effect of porcine placenta extract on HaCaT cell viability.

## Appendix A

To investigate the effect of porcine placenta extract on human skin, we estimated how its application influences the expression of ceramide synthase (*CERS*) in human keratinocyte cells (HaCaT).

### Appendix A.1. Materials and Methods

#### Appendix A.1.1. Cell Culture

HaCaT, Human keratinocyte cell line, were incubated and maintained in Dulbecco’s Modified Eagle Medium (DMEM) high glucose (Wako, Japan) supplemented with 10% fetal bovine serum (FBS) (GE Healthcare HyClone, USA) and 1% penicillin-streptomycin (PS) (Wako, Japan) solution. The cells were incubated in a humidified atmosphere containing 5% CO_2_ at 37 °C.

#### Appendix A.1.2. MTT Assay for Cell Viability

To assess the cell viability, HaCaT cells were seeded in a 24-well plate at a density of 1.0 × 105 cells/well at 37 °C under humidified air containing 5% CO_2_. After 24 h, porcine placenta extracts or solvent (Water) were added to the incubated cells. Then, 48 h later, fresh media and 3-(4,5-Dimethyl-2-thiazolyl)-2,5-diphenyltetrazolium Bromide (MTT) solution were added to each well. Incubation was continued for another 4 h. After incubation, the culture media was discarded, and 40 mM HCl-isopropanol added to each well. The absorbance was measured at 570 nm using a microplate reader.

#### Appendix A.1.3. RT-PCR Analysis

To estimate the expression of ceramide synthase genes, HaCaT cells were collected at 48 h after treatment of the porcine placenta extracts. Total RNA was extracted using a RNeasy Mini kit (Qiagen, USA) and cDNA was synthesized using ReverTra Ace qPCR RT Master Mix with gDNA Remover (Toyobo Co., Ltd., Japan). RT-PCR was performed using the THUNDERBIRD SYBR qPCR Mix (Toyobo Co., Ltd., Japan) for CERS2, CERS3, CERS4, and β-actin (which was used as the internal control). PCR was performed with primers as follows: 5’-CCGATTACCTGCTGGAGTCAG-3’ and 5’-GGCGAAGACGATGAAGATGTTG-3’ for CERS2, 5’-ACATTCCACAAGGCAACCATTG-3’ and 5’-CTCTTGATTCCGCCGACTCC-3’ for CERS3, 5’-GGAGGCCTGTAAGATGGTCA-3’ and 5’-GAGGACCAGTCGGGTGTAGA-3’ for CERS4, 5’-GGGTCAGAAGGACTCCTATG-3’, and 5’-GTAACAATGCCATGTTCAAT-3’ for β-actin. The 2−ΔΔCt method was used to calculate the relative mRNA levels. The PCR was performed by using an Agilent AriaMX Real-Time PCR system (Agilent Technologies, USA).

### Appendix A.2. Result and Discussion

The expression of the *CERS2* gene in placenta extract-treated cells did not differ significantly from that in control cells. However, *CERS3* and *CERS4* levels in placenta extract-treated cells were higher than those in control cells. *CERS3* and *CERS4* expression was similar among cells treated with different concentrations of porcine placenta extract (Figure A1). In addition, the viability of HaCaT cells after the application of porcine placenta extract was not significantly different from that of control cells (Figure A2). These results suggest that porcine placenta extract has the potential to enhance the expression of *CERS3* and *CERS4* genes and exhibits no cytotoxicity toward HaCaT cells. Therefore, porcine placenta may be expected to promote the synthesis of ceramide, which plays an important role in human skin barrier function.
